# ‘Old Is Gold’: How Traditional Indian Dietary Practices Can Support Pediatric Diabetes Management

**DOI:** 10.3390/nu13124427

**Published:** 2021-12-10

**Authors:** Sheryl Salis, Anju Virmani, Leena Priyambada, Meena Mohan, Kajal Hansda, Carine de Beaufort

**Affiliations:** 1 Department of Nutrition, Nurture Health Solutions, Mumbai 400098, India; 2 Department of Pediatric Endocrinology, Max Super Speciality Hospital, New Delhi 110017, India; virmani.anju@gmail.com; 3 Department of Pediatric Endocrinology, Madhukar Rainbow Children’s Hospital, New Delhi 110017, India; 4 Department of Pediatric Endocrinology, Pentamed Hospital, Delhi 110009, India; 5 Division of Pediatric Endocrinology, Rainbow Children’s Hospital, Hyderabad 500034, India; leenapriyambada@gmail.com; 6 Department of Pediatric Endocrinology, PSG Super Speciality Hospital, Coimbatore 641004, India; meenapaed@gmail.com; 7 Department of Nutrition, Diabetes Awareness and You, Kolkata 700039, India; day.kajalh@gmail.com; 8 Department of Pediatric Endocrinology, DECCP/Centre Hospitalier de Luxembourg, 1210 Luxembourg, Luxembourg; bcschim@pt.lu; 9 Faculty of Science, Technology and Medicine, Université of Luxembourg, 4365 Esch-sur-Alzette, Luxembourg; 10 Department of Pediatric Endocrinology, Free University Hospital Brussels UZ-VUB, 1090 Bruxelles, Belgium

**Keywords:** diabetes management, medical nutrition therapy, traditional Indian practices, ancient food wisdom, India, glycemic control, diabetes education, Indian *Thali* concept

## Abstract

Nutrition is crucial for maintaining normal growth, development, and glycemic control in young people with diabetes (PwD). Undue restrictions cause nutrient deficiencies as well as poor adherence to meal plans. Widespread availability of low-cost, ultra-processed, and hyperpalatable food is further damaging. Most families struggle to find ways to provide nutritious, yet attractive, food with a low glycemic index (GI). India is one of the oldest continuous civilizations with a rich and diverse cultural and culinary heritage. Traditional dietary practices, including the centuries-old ‘*Thali*’ (meaning plate) concept, emphasize combinations (grains, lentils, vegetables, dairy, spices, prebiotics and probiotics, and fats) of local, seasonal, and predominantly plant-based ingredients. These practices ensure that all of the necessary food groups are provided and fit well with current evidence-based recommendations, including the International Society for Pediatric and Adolescent Diabetes (ISPAD) 2018 Guidelines. Techniques for the preparation, cooking, and preservation of food further impact the GI and nutrient availability. These practices benefit nutrient density, diet diversity, and palatability and thus improve adherence to meal plans and glycemic control. This narrative review describes the ancient wisdom, food composition, and culinary practices from across India which are still valuable today. These may be of benefit worldwide to improve glycemic control as well as quality of life, especially in PwD.

## 1. Introduction

India is one of the oldest continuous civilizations with diverse religions, cultures, traditions, socioeconomic strata, and agricultural practices living in harmony for millennia. The highly varied geography includes mountains, plains, deserts, tropical and subtropical forests, as well as a long coastline; from this geographical diversity comes an extensive biodiversity in plant species and food traditions [1,2,3]. An important aspect of Indian tradition is the holistic approach to health and culinary practices, which is aimed at overall wellness, centuries before health was defined as a “state of complete physical, mental and social well-being and not merely the absence of disease or infirmity” [4].

Diabetes, particularly type 1 diabetes, forces families to pay attention to food. Meal plans, timings, and discipline are key components in managing the intricate balancing act of a good glycemic control. The 2018 International Society for Pediatric and Adolescent Diabetes (ISPAD) Clinical Practice Consensus Guidelines recommend a diet “based on healthy eating principles suitable for all children and families, with the aim of improving diabetes outcomes and reducing cardiovascular risk” [5]. Post-prandial hyperglycemia interferes with tight glycemic control and contributes to the development of chronic complications. With increasing commercialization and availability of hyperpalatable foods with high glycemic index (GI), families struggle to ensure nutritious, yet attractive meals [6]. Parents also seek ways to vary dietary options for fussy children and adolescents. Further, type 1 diabetes is associated with a higher risk of celiac disease, which requires a gluten-free diet, considerably complicating dietary management. Working on the premise that people with diabetes (PwD) should have a healthy, balanced meal plan and that there is no special “diabetic diet” [7], this narrative review explores the benefits of traditional food practices from across different parts of India, with a special emphasis on low GI foods which will be useful in type 1 as well as type 2 diabetes. The paper draws upon experience of traditional common usage, supported by available literature retrieved from various databases (PubMed, Google Scholar, and relevant Internet official websites) as cited, using relevant keywords related to the topics discussed.

## 2. How Did Ancient India Manage a Healthy Diet?

A recent report of an excavation in a northwestern Indian state revealed multigrain, high-protein, handmade sweet balls (*ladoo)* prepared from wheat, barley, chickpea, and oilseeds from the 4000-year-old Harappan civilization [8], showing that this ancient culture had an understanding of balanced nutritional composition. 

The Vedic scholars developed the science of Ayurveda between 2500–500 BC for managing a healthy lifestyle [9]. Ayurveda comes from two words: *ayus*, meaning life and *veda*, meaning study or knowledge; hence *‘Ayurveda’* means knowledge of life. It not only encompassed detailed medical science, but also emphasized nutrition, exercise, and other aspects to promote physical, as well as mental, wellbeing, strengthen immunity, and enable effective gut function [10]. Food practices focused on function and flavor. The key principles included individualization to match the elements of existence, body types, professions, and the local and seasonal availability of ingredients, all while minimizing waste. Food would be season-specific (‘warming’ foods in winter and vice versa), based on the Ayurvedic concept that there is ‘nature’s wisdom’ in what is seasonally available [10]. Ideally, meals were supposed to have all six tastes: sweet, sour, salty, pungent or spicy, bitter, and astringent. The ancient culture realized that multiple constituents like herbs, spices, and other food components may work synergistically to produce a therapeutic effect [11]. Traditionally, people in India sat down comfortably on the ground to have their meals along with the entire family. It was believed that this ritual improves bonding with the family as well as helps in digestion [10].

A traditional meal was mostly plant-based, consisting of grains (cereals, millets), pulses [12,13], a variety of spices (like pepper, cumin, coriander, and ginger), local seasonal vegetables and fruits, as well as a milk-based product (yogurt, buttermilk, and cottage cheese) to meet the daily energy, macronutrient, micronutrient, fiber, and antioxidants requirements. Eggs, free range poultry, fish, and meat were featured in the diets of non-vegetarians, but in limited amounts. Cooking was done with cold-pressed oils (mainly groundnut, sesame, mustard, or coconut, depending on the region) and clarified butter (*ghee*), prepared from milk, to provide energy and to improve both palatability and the absorption of fat-soluble nutrients [14]. Meals were balanced not just in terms of nutrition, but also in terms of taste and texture. Apart from freshly prepared food, several ways of processing food for storage and preservation were used as refrigeration was not available. Natural, home-cooked meals were consumed daily, while elaborate menus of rich, refined, high-fat dishes were reserved for festive occasions. The plethora of functional foods offered the whole range of necessary nutrients (antioxidants, polyphenols, fibers, natural prebiotics and probiotics, and other bioactive compounds). Processing techniques such as sprouting, malting, and fermentation enhanced nutrient content [2], helped to stimulate digestion and assimilation, and conferred other health benefits. Many of these traditional practices are followed in India even today. Table 1 explores some of the cooking methods and techniques used in Indian cooking which add to the nutritive value of food and/or lower the GI or glycemic load (GL) of food.

Over the centuries, with travel and migration, ‘foreign’ plants and ingredients were assimilated into local Indian cuisines. There were innovations, intermingling, and adaptation of food traditions.

The ‘Plate Method’ has been in practice for centuries as ‘*Thali*’ [14]; *Thali* means plate in many Indian languages. The principle of *Thali* is to provide nutritionally balanced meals in a variety of textures and flavors with portion control by using small bowls for each food. Foods from all food groups, including whole grains, vegetables, pulses or non-vegetarian items, and a dairy product are incorporated.

**Figure 1:**
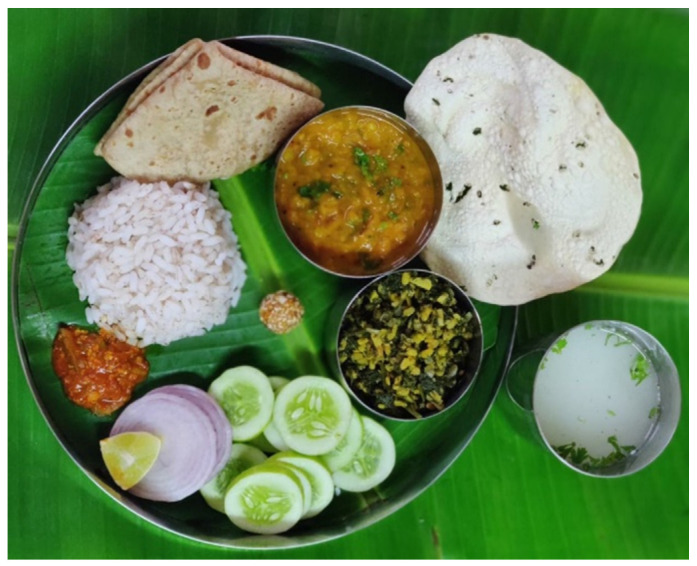
Typical Indian *Thali. Thali* consisting of whole wheat *chapati*, brown rice, lentil curry, green vegetable with split yellow lentil, cucumber slices, onion slices, lemon wedge, pickle, thin, spiced, roasted disc made of pulse (*papad*), buttermilk with herbs, and a sweet (sesame *ladoo*), served on a banana leaf. This original picture is representative of a regular, homemade Indian *Thali* prepared by author S.S.

Traditional Indian *Thali* meals included all food groups, providing a balance of proteins, fats, carbohydrates, dietary fiber, and a plethora of phytochemicals by incorporating a variety of plant foods in different colors [1,2,3,32]. The emphasis was on using locally and seasonally available ingredients. A preponderance of vegetarian food items was common, even amongst non-vegetarians, though coastal and riverine areas used fish and other seafood liberally. Figure 1 depicts an example of a regular Indian vegetarian *Thali* meal.

The Indian *Thali* matches well with the ISPAD 2018 recommendations “children and adolescents with diabetes should eat a variety of healthy foods, including fruits, vegetables, dairy, whole grains, legumes and lean meat in amounts appropriate for age, stage of growth and energy requirements” [5].

## 3. Medical Nutrition Therapy in Type 1 Diabetes

Medical nutrition therapy (MNT) forms one of the pillars for the management of type 1 diabetes [5]. The goals of MNT are to ensure normal growth and development, sustain adequate physical activity, maintain normal body mass index (BMI) for age and gender, maintain good glycemic control, and help prevent short-term and long-term complications. The meals should be balanced with the necessary micronutrients and macronutrients, adequate fiber, and fluids. The medical nutritional advice should address the child’s needs and the family’s cultural, social, and personal preferences. To optimize glycemic control in PwD, the focus should be on achieving a balance between food intake, metabolic requirements, energy expenditure, and insulin action profile. The ISPAD 2018 Guidelines recommend that carbohydrates should provide 45–50% of calories, fats < 35% (saturated fat < 10%), and proteins 15–20% [5]. Families should be encouraged to eat the same food.

The primary determinants of the dose of the pre-meal insulin bolus are the amount and type of carbohydrates in the meal, the GI, and GL. Low GI foods are preferred, except when a rapid rise in blood glucose (BG) is desired (e.g., exercise and hypoglycemia correction). Choosing low to moderate GI and GL foods over high GI and GL foods [33], maintaining consistency in carbohydrate quantity and quality, and matching insulin to carbohydrate intake help achieve better glycemic control [34]. Low GI (<55) and low-to-moderate GL (<20) dietary patterns have also been shown to improve the lipid profile in individuals with moderately controlled type 1 and type 2 diabetes [35].

Although low carbohydrate diets are sometimes attempted to improve glycemic control, they can be nutritionally inadequate, impair growth and development, increase the risk of disordered eating behaviors, increase hypoglycemia or potentially impair the effect of glucagon in hypoglycemia, and increase cardiovascular risk [5,17,36,37]. Protein and fat intake also need to be considered for calculating the insulin dose as they impact insulin needs [38,39] and alter the rate of absorption of carbohydrates. In those who have concomitant celiac disease, the meals must also be free of all traces of gluten. Maintaining optimum weight and preventing obesity is equally important for maintaining good glycemic control, which is most successfully done with a family-centered approach [5].

The increasingly popular hyper-palatable, ultra-processed, commercial foods, with high sugar, salt, and saturated and total fat content, have caused a sharp rise in the prevalence of obesity, type 2 diabetes, hypertension, and other non-communicable diseases [6,40,41]. People with type 1 diabetes are equally susceptible to these problems. The diagnosis of diabetes may be an opportunity for the entire family to improve eating habits and, therefore, their overall health.

## 4. Essential Components of Indian Traditional Nutrition

### 4.1. Carbohydrates

Carbohydrates provide 65–70% of calories across all regions in India, as shown in the starch study [42]. Rice, wheat, maize, various millets, amaranth, barley, starchy vegetables, fruits, and added sugars are the predominant sources of carbohydrates. Pulses and milk also contain carbohydrates. Typically, an Indian meal is a combination of grains, pulses, vegetables, and dairy, with or without a non-vegetarian item [43]. These carbohydrate-protein-fiber combinations ensure that all essential amino acids and micronutrients are provided in every meal [43].

### 4.2. Dietary Fiber

Soluble fiber is an essential ingredient for healthy nutrition [44,45]. The high fiber content of Indian meals comes from vegetables, fruits, whole grain cereals, millets, and whole pulses. Vegetables are a part of meals in multiple ways: as individual dishes; added to grains, pulses, or in non-vegetarian dishes; as salads, accompaniments, or spiced pastes (*chutneys*); or mixed with curd (*raita*). Some vegetables are pickled (e.g., turnips, carrots, or black carrots), or dried (e.g., mint, fenugreek, or moringa leaves), for later use.

A wide variety of seasonal, green, leafy plants such as spinach, fenugreek, amaranth, and mustard leaves are used. These provide iron, calcium, vitamins, antioxidants, and fiber without increasing the calorie and carbohydrate content [46]. The leaves of some plants are also used for wrapping food as a part of the cooking process, such as banana, turmeric, mantharai, jackfruit, colocasia, koupat, and Alpinia leaves. The cooking technique could be steaming, roasting, grilling, or frying. The leaves provide an effective casing, protecting the food from being exposed to direct heat; they trap steam and seal in the flavors, allowing food to cook slowly on a low flame, marinating in its own juices [27]. This minimizes the oil needed for cooking while increasing the polyphenol and antioxidant content [47]. The leaves also add variety and flavor to the meal.

The abundance of leaves, fresh fruits, and vegetables provide fiber and micronutrients to the daily diet. These ideas can be incorporated in the cooking repertoire for children with diabetes and/or celiac disease (as they are gluten-free), making vegetables more acceptable and palatable.

### 4.3. Lente Carbohydrates

Lente carbohydrates are slowly absorbed in the body as they contain high amounts of viscous fiber, thereby delaying gastric emptying time and blunting the post-meal BG response. Sources of lente carbohydrates are lentils, split grams, chickpeas, kidney beans, green peas, soaked pulses and barley, which are commonly consumed in Indian cuisine [48,49].

### 4.4. Resistant Starch

Many starchy foods have a high GI, causing post-meal hyperglycemia in people with type 1 diabetes. Resistant starch (RS), present in a variety of starchy foods, acts like fiber in the large intestine as it is not digested in the small intestine. Hence, RS blunts the glycemic response, apart from having prebiotic benefits. Several traditional Indian preparations and whole-grain products such as cereals, legumes, nuts, seeds, and starchy vegetables contain high amounts of RS. RS type 1 (RS1) is a physically inaccessible starch found in partially milled grains, legumes such as beans or lentils, and seeds [20]. Unripe plantains, plantain flour [50,51,52], tender jackfruit and its flour, and raw potatoes are naturally rich in RS type 2 (RS2) [53]. Cooking, cooling, and then gently reheating high GI starches (rice, potatoes, sweet potatoes, or pasta) increases the RS content. Hence, care must be taken not to heat RS much, as it gets degraded at high temperatures [51].

### 4.5. Sources of Carbohydrate

#### 4.5.1. Rice

Rice is the predominant cereal in southern, eastern, and north-eastern India [54], with thousands of varieties grown across the country. Being gluten-free, it is suitable for those with celiac disease.

The preparation and preservation of rice have an important impact on its GI and need to be considered to maintain good glycemic control.

The rice kernel consists of four parts: the outermost inedible husk, bran, endosperm, and embryo. The bran contains fiber, vitamins, iron, fat, anthocyanin (pigment), non-starchy polysaccharides, and polyphenols. The endosperm is predominantly starch and encloses the embryo. Milling removes the husk and part of the bran; polishing further removes more bran and yields white rice [23,55]. Current technology enables efficient milling and polishing, producing attractive white rice. This product, however, has a much higher GI of 70–77 versus 57 of the traditional hand-pounded and winnowed brown rice. Increased use of this white rice may have contributed to the rise in obesity and type 2 diabetes, particularly in South Asia [56].

The grain structure of rice also has an impact on the GI; unpolished rice with bran, long grain basmati rice, parboiled rice, and brown rice have a lower GI than short-grain rice, milled, or polished white rice [17,57].

Brown rice is a good source of magnesium, phosphorus, selenium, thiamine, niacin, and vitamin B6 [58]. Other varieties of colored rice (red rice, black rice) get their color from the anthocyanin in the bran, which imparts free radical scavenging and antioxidant properties [59].

A traditional rice preparation method is parboiling: a hydrothermal treatment that involves soaking the paddy in water, heating, drying, and then milling. This causes gelatinization of the starch into tightly packed structures, resulting in harder and glassier-looking kernels, and a much lower GI [55,60,61,62,63]. The parboiling process preserves the endosperm micronutrients (vitamins and minerals) contained in the bran, which are usually removed in the whitening process in the rice mill.

Traditionally, old (i.e., stored and aged) rice was preferred to freshly harvested rice. The aging of rice increases the peak viscosity, making starch granules more resistant to swelling and rupture during cooking, thereby lowering the GI [64].

A further reduction in GI is observed after refrigerating cooked rice for 24 h and reheating at low temperatures. This is due to the process of retrogradation, which increases resistant starch type 3 (RS 3) content [21]. The cooked rice that was stored overnight [21] is sautéed on a low flame with added vegetables, tamarind *chutney,* and peanuts, which further blunt the GI and make it a wholesome meal. This would be particularly beneficial to PwD, even more so if accompanied by a bowl of plain yogurt or *raita* [17].

Fermentation is frequently used across the country for different foods. A popular dish was cooked rice that is then soaked in water and allowed to ferment overnight. The superfluent water with added salt is an excellent hydrating agent in the hot and humid summers, while the fermentation improves the bioavailability of iron, potassium, and calcium by several folds, providing B-complex vitamins, especially B12, and probiotics [28]. The resistant starch (it was not heated the next day) reduced the GI. Rice, in combination with locally available pulses, vegetables, and sometimes yogurt or yogurt-based preparations, tempered with different spices, yielded a vast array of dishes.

Box 1 summarizes some traditional ways to reduce the GI of rice-based meals.

Box 1 **Traditional ways to lower glycemic index and improve the nutritive value of rice-based meals.**

- Using hand-pounded rice: brown/red/black rice
- Using parboiled rice instead of polished white rice
- Using old (i.e., stored and aged) rice
- Using cooked rice that has been cooled overnight
- Combining rice with protein sources like pulses, yogurt, cottage cheese, egg, fish, poultry, meat
- Adding *ghee* or nuts and seeds in moderate amounts
- Combining rice with a variety of vegetables as a part of a mixed meal
- Squeezing lemon or adding tamarind to rice meals

#### 4.5.2. Wheat

Wheat is the staple grain in northern and central India. Whole wheat contains complex carbohydrates, dietary fiber, B vitamins, phytochemicals, and some minerals. Milling of wheat into all-purpose flour (refined flour), or even semolina, destroys the intact cell wall structure in which the starch granules are entrapped and increases the surface area, thereby exposing the starch to enzymatic digestion and increasing the GI. Nutrients are also lost when the husk is removed after milling. Therefore, the consumption of food made from whole wheat flour is recommended over refined flour-based products [15,18,59,65,66], especially in diabetes.

Wheat is still widely consumed in India as homemade unleavened flatbread—*roti* or *chapati*—made of whole wheat flour, despite increasing use of refined carbohydrates like polished white rice, or refined flour products in industrial foods. The *chapati* has a GI of 45–72 [17,67], while the GI of white bread varies from 63–93 [68], due to the 75% loss of fiber during milling. A vast variety of preparations evolved from the basic *chapati* by adding pulses, vegetables, eggs, meat, or spices, as well as by cooking them in different ways. These modifications are described in detail in Supplementary Table S1.

Apart from being ground into whole wheat flour, wheat is sprouted and made into porridge. Sprouting for a limited period increases the activities of hydrolytic enzymes, improves the content of some essential amino acids and vitamin B, and decreases dry matter, starch, and anti-nutrients [25]. The partial hydrolysis of storage proteins and starch during sprouting improves their digestibility [69,70].

Emmer or *‘Khapli’* wheat (*Triticum dicoccum*), one of the world’s ancient cereals, was among the first cereals to be grown in the fertile plains of India and is being revived. Compared to polished wheat, it is more disease-resistant, has higher dietary fiber (almost 9–20%), amylose, protein and ash content, has a lower fat and carbohydrate content, and therefore has a lower GI [71,72].

Box 2 summarizes some practical ways to lower the GI and improve the nutritive value of wheat-based meals [17].

Box 2 **Traditional ways to lower glycemic index and improve the nutritive value of wheat-based meals.**

- Using whole wheat flour and *Khapli* (Emmer) wheat
- Using broken wheat and larger grit semolina
- Mixing bran and pulse flours like gram flour, soya flour, or millet flour with whole wheat flour
- Adding grated or pureed vegetables, green leafy vegetables, herbs, and spices to whole wheat flour
- Kneading whole wheat flour with yogurt, whey, milk, leftover pulse curry, or vegetable curries
- Stuffing the *chapati/paratha* made from whole wheat flour with protein sources such as egg, minced meat, cottage cheese, pulses, and vegetables
- Adding fat in moderate amounts

#### 4.5.3. Barley

Barley is mentioned in the Rigveda (4000–8000 years ago) as a staple food, predating wheat. It has a low GI of 25 [15] and is rich in β-glucan (a soluble fiber that decreases serum cholesterol and post-prandial BG levels), protein, and vitamin B [42]. It is consumed as flatbread and porridges. Barley reduces the GI of the *chapati* when mixed with whole wheat flour [73].

#### 4.5.4. Millets

Wheat and rice were grown and consumed mostly in the fertile northern Gangetic plains. However, large parts of the subcontinent were arid or semi-arid and relied on a variety of millets as their staple grain. Some of the better-known millets are finger millet, pearl millet, sorghum, barnyard millet, foxtail millet, kodu, proso millet, and little millet. The consumption of millets is gaining prominence today.

Millets are hardy plants with a short growing season of 45–60 days, can grow in poorer soil, need much less water or pesticides than cereals, and contain more protein, fiber, phytochemicals, antioxidants, and minerals [74]. The pectin, cellulose, and hemicelluloses in their seed coat, which are resistant to breakdown by digestive enzymes, make them sources of fiber. They are richer in amino acids (especially the sulfur-containing amino acids, methionine and cysteine), calcium (especially finger millet), polyphenols, and fatty acids as compared to maize and rice [75].

Pearl millet, a staple in the western part of India, has a GI of 55 [75] and is an important dietary source of iron and zinc [76,77]. Millets are effective substitutes for rice and wheat in recipes such as flatbreads, crepes, dumplings, *idlis*, pancakes, soups, salads, and other dishes. Most millets have lower GI than wheat as well as rice, and thus beneficial in diabetes. However, finger millet is an exception, with a high GI of 84 [78]. Being gluten-free, millets are useful for people with celiac disease.

Supplementary Table S1 provides traditional Indian preparations using whole grains and cooking methods that lower the GI and enhance the nutritive value of the meal.

#### 4.5.5. Maize

Maize was introduced in India as late as the 16–17th century by Portuguese traders and is now grown across the country. Rich in magnesium and other minerals [77,79], the dried corn is milled into a coarse flour and made into moderately thick flatbreads (northern India), broken coarsely to be made into porridge (central India), consumed with vegetables, or simply roasted or boiled, and eaten off the cob, with a rub of lemon juice to lower the GI [80]. In these forms, it has an acceptable GI of 40–55, in contrast to a GI of 75–95 of commercially produced cornflakes or high-fructose corn syrup [17,81].

#### 4.5.6. Starchy Vegetables

Starchy vegetables, such as potatoes, sweet potatoes, corn, yams, or taro root (colocasia) have become popular in Indian cooking in the last 500 years. The carbohydrate content of these vegetables must be factored in for insulin dosing. Two popular starchy vegetables with prebiotic properties and moderate GI, which are native to India, are raw banana (plantain) and tender jackfruit [52,53,81,82]. Plantain and jackfruit flours are useful for supporting smooth glycemic control in PwD and for celiac disease.

Bananas have been considered sacred and are part of many religious ceremonies in India. All parts of the plant are used: the raw and ripe fruit, the flowers, and the pith are eaten; the leaves are used to wrap food for cooking and to serve food. They are a good source of resistant starch [20,27,50,51] as well as fiber, potassium, magnesium, and vitamin B6. Raw bananas can be used as flour, or as vegetables in curries, fries, stews, and soups. They can be used as a replacement for potatoes. Depending on the variety, the GI may differ between 45–70, which needs to be considered when adjusting insulin treatment [81,83].

Another native plant is jackfruit. Tender jackfruit is a delicacy in India. Apart from being prepared as vegetables, curries, and pickles, its flour is also used as a cereal substitute due to its fiber and resistant starch content, lower GI and high nutritive value when compared to wheat and rice [53,82,84,85].

#### 4.5.7. Pseudo Cereals

Pseudocereals, such as buckwheat, amaranth, and bamboo rice, have been used since ancient times, particularly on holy days as cereal substitutes. Buckwheat, native to the Himalayas, has a high fiber content (27.4%) that is closer to vegetables than other cereals. There is also a high proportion of resistant starch (33.5–37.8%) and proteins with a well-balanced amino acid composition. It also contains minerals including zinc, copper, and manganese; bioflavonoids, especially rutin and quercetin; and high antioxidant activity. With a GI of about 50 [81,86], it is an excellent food for managing diabetes. Amaranth has high protein content (13.6 gm), with relatively high lysine, calcium, and fiber. However, it also has a high GI [87]. Pseudocereals have the added benefit of being gluten-free.

#### 4.5.8. Added Sugars

ISPAD 2018 guidelines recommend that sucrose use should be minimized in diabetes and can provide up to 10% of total daily energy intake [5]. This may help improve the acceptability while planning menus for children with type 1 diabetes.

A wide range of healthy traditional dessert options, with variable degrees of sweetness, evolved in different regions. They could have a base of pulses, nuts, appropriate dry fruits, fruits, or vegetables. For example, sweetened balls *(ladoos)* are made from sesame, peanuts, Bengal gram, green gram, black gram, and other seeds. Many had milk or milk-based products as the base ingredient, such as desserts made of thickened milk, low-fat hung curd, and cottage cheese [1,2,88].

Sugars may be present in *chutneys*, pickles, and preserves in significant quantities and can give rise to unexpected glucose spikes. In some cuisines, sugar or jaggery is added to all gravies and vegetables, which needs to be factored in when counting carbohydrates [89].

In summary, it is possible for PwD to get 45–50% of their energy from carbohydrates and achieve good glycemic control by switching to whole grains, especially unpolished millets, that provide low to moderate GI complex carbohydrates with resistant starch, high fiber, and micronutrients.

### 4.6. Proteins

India has the highest number of vegetarians (20–30%) in the world [90] and even most ‘non-vegetarians’ usually do not consume non-vegetarian preparations more than a few times in a week. Implemented intelligently, vegetarian meals are nutritious and meet good quality protein requirements well [91,92]. Children and adolescents with diabetes (type 1 or type 2) need the same amount of protein as their peers without diabetes. Approximately 15–20% of energy should come from protein [5]. Traditional Indian meals ensured good quality proteins in vegetarian diets by combining cereals with pulses, nuts, and seeds with an emphasis on the consumption of low-fat dairy products [90,93,94].

#### 4.6.1. Pulses

Whole pulses have less carbohydrates and twice the amount of protein than whole-grain cereals (30 g pulse provides 7 g protein, and 15 g carbohydrate). The carbohydrate is mainly complex and non-digestible (soluble and insoluble dietary fibers, resistant starches and oligosaccharides), and is hence a “good” (low GI) carbohydrate [95]. Pulses are also a source of unsaturated fat, essential micronutrients, and bioactive phytochemicals, mainly polyphenols and phytosterols. These lente carbohydrates, with their low fat and high fiber content, help provide satiety and stabilize BG levels [15], making them nutritious and beneficial for PwD.

There is a wide variety of pulses grown in India, and of recipes using them. They can be easily integrated into food practices across the world, improving glycemic control, protein and fiber content, and overall nutritive value and palatability. The most popular pulses include pigeon pea, green gram, Bengal gram, chickpea, black gram, moth beans, lentil, cowpea or black-eyed beans, horse gram, peas, and kidney beans. These whole or split pulses (with or without skin) are a part of major meals. They are usually soaked, boiled, and tempered with spices or curry leaves cooked in a little oil or *ghee*. Soaking before cooking reduces cooking time, improves digestibility, and reduces anti-nutritional factors like phytates, tannins, and enzyme inhibitors. This increases the absorption of the nutrients and reduces the bloating effect of the lectins and phytates [15,24,25]. Sour additives such as *Kokum (Garcinia indica),* tamarind, raw mango, gooseberry, or lemon to the pulses are common and lower the GI while adding nutritive value and taste to the dishes [80].

Pulses are often cooked with vegetables and/or sometimes meat. Many pulses can be sprouted (germinated) and then eaten raw, steamed, or pureed to make pancakes. Sprouting furthers the advantages of soaking, increasing the bioavailability of protein and micronutrients (folate, magnesium, phosphorus, manganese, and vitamins B12, C and K) [25].

Soybeans (Glycine max) are particularly rich in proteins: 100 g has 36 g protein [77], equivalent to almost 5 eggs while being low in carbohydrates.

For pulses, soy, and other beans, it is important to consider the increase in GI with increased processing (blending, grinding, milling, and pureeing) and smaller particle size [96]. Insulin doses should be adjusted accordingly.

Supplementary Table S2 describes some common Indian preparations using pulses, and Supplementary Table S3 has cereal-pulse combinations, which lower glycemic index (GI) and enhance the nutrient value of meals.

Some commonly used ways to include more pulses in the meals are presented in Box 3.

Box 3 **Traditional ways to improve the nutritive value of meals using pulses.**

- Using whole pulses or pulses with skin
- Using sprouted pulses (raw/steamed/ground/pureed)
- Adding pulses (and/or its flours) like soybean to grains like wheat, rice, and millets in meals and snacks
- Adding pulses to vegetables and meat curries
- Substituting cereals with pulses in snacks and meals e.g., in pancakes, roasted gram
- Consuming pulse-based spiced pastes (*chutneys*) with meals

#### 4.6.2. Dairy Products

The consumption of dairy products has been traditionally encouraged across the country. Milk was consumed in various forms and was also used for obtaining fat (*ghee*) for cooking. Apart from being consumed plain, healthy additives like powdered nuts or spices improve the taste and nutritive value. Adding raw turmeric powder to milk was traditionally popular for enhancing immunity against infections and improving sleep quality; this is now popular globally as Golden Milk or Turmeric Latte. Curcumin is also known to be beneficial in type 2 diabetes [97].

Buttermilk and fresh, homemade yogurt using live cultures, were part of all major meals and contributed proteins and other nutrients to meals. Yogurt and buttermilk are easy to digest (even by lactose-intolerant individuals) [98], provide hydration, and offer probiotic benefits. Yogurt-based drinks could be watery or thick and could be plain, salty, or spiced buttermilk; these drinks are low or moderate in calories. They are considered “cooling beverages”, which are useful in the hot summer, and before or after exercise.

Indian cottage cheese/soft cheese (*paneer*), containing 20 g proteins, 12 g carbohydrate/100 g, is a very popular food item which has been used in a wide variety of ways [77,99].

Milk, yogurt, buttermilk, and paneer are excellent options for meal planning in diabetes, particularly type 1 diabetes. They can be between meal or bedtime snacks or drinks, as they have low carbohydrate content (4 g in 100 g of plain yogurt). Consuming milk in recommended amounts at bedtime can prevent nocturnal hypoglycemia, especially in PwD using regular and intermediate-acting insulins at night [77].

#### 4.6.3. Eggs, Fish, Poultry and Meat

Fish and other seafood are consumed regularly in the coastal areas and provide essential fatty acids and good quality protein. The meat most widely available is goat meat (mutton) and chicken, which is less fatty than beef, pork, or lamb meat [100]. Being negligible in carbohydrates and high in protein, they have a limited impact on insulin doses and BG values in PwD when consumed in moderation. Eggs are a preferred choice as mid-meal snacks for children on rapid or fast-acting insulin.

### 4.7. Fats and Oils

It is important to have adequate fats in the diet, particularly for the growing child, to provide palatability, energy, and essential fatty acids especially fat-soluble nutrients. In PwD, the added fats also reduce the GI of the meal. However, in excess, fats impair glycemic control and cause obesity and dyslipidemia, increasing the risk of cardiovascular disease. The American Heart Association advises children to be given more polyunsaturated (PUFA) and monounsaturated (MUFA) fatty acids than saturated fats to reduce cardiovascular risk later in life [101]. ISPAD 2018 Guidelines recommend replacing saturated fat with unsaturated fats by using lean meats, fish, low-fat dairy products, and changing to MUFA and PUFA cooking oils. Consumption of 80–120 g of oily fish (rich in n-3 fatty acids) is recommended once or twice a week [5].

Traditionally, the fat sources in Indian cooking were edible vegetable oils, *ghee*, and milk products, with a small percentage coming from meat and fish [102]. The vegetable oils—mainly mustard, groundnut, sesame, and coconut oil—were mechanically pressed, preserving not only the fatty acid components and functional compounds, but also the flavor and overall quality of the oil. These traditional oils need to be re-emphasized as the medium for cooking as they have a good MUFA content. Mustard oil, with high n-3 PUFA content and medicinal properties, was used in daily cooking and to make a variety of pickles. Many of these nutrient-rich pickles (made from gooseberry, garlic, raw mango, and mixed vegetables) are easily prepared. Eaten in small quantities with steamed foods, they add micronutrients, beneficial probiotics and piquancy to meals.

Coconut oil is often vilified for its 91% saturated fatty acid content; similarly, clarified butter (*ghee*) is condemned as being a saturated fat. However, they are less atherogenic than animal and other plant fats like lard and *vanaspati* [103]. The traditional adding of *ghee* to *chapatis*/steamed rice/*idlis*/dals flattens post-meal glucose spikes, apart from adding taste and aroma to the meal [66]. Since certain dishes were meant to be prepared in specific oils, it was easy to use a combination of fats, and thus meet the requirements of all the essential fatty acids for complete nutritive value and cardiac health [102].

Marine fish such as salmon, sardines, or *hilsa*, which are particularly rich in eicosapentaenoic acid (EPA) and docosahexaenoic acid (DHA), are consumed in coastal areas [16].

Though carbohydrates are the primary determinants of insulin doses, fat and total calories must also be considered when calculating pre-meal doses. It is easier to reduce post-meal spikes and enhance satiety by consuming some healthy fat in each meal.

#### Nuts and Seeds

A variety of nuts, such as cashews, almonds, walnuts, sesame, pistachio, and pine nuts, as well as the less expensive seeds, including melon, watermelon, pumpkin, cucumber, flaxseed, and peanuts, are commonly used. Roasted peanuts or foxnuts, and other seeds, can be eaten by themselves, mixed with other nuts, or added to gravies, salads, rice, drinks, tangy *chutneys*, or desserts. They are low in carbohydrates, and are a good source of MUFA, protein, fiber, and antioxidants. Some nuts like walnuts and flax seeds are good sources of omega 3 fatty acids [104]. Peanuts (‘poor man’s protein’) can be roasted, fried, or steamed and are very popular as snacks or added to dishes, enhancing protein and healthy fat content. Adding whole or crushed roasted peanuts to vegetables and cereal-based savory grain items, or having peanut *chutney* with *idli* or *dosa*, as is commonly done, lowers the GI [105]. Many preparations use fresh coconut, which adds fiber, medium-chain triglycerides, and micronutrients [106]. This is a low carbohydrate snack, but high in fat and calories, and hence can be consumed only in moderation.

The range of low GI snacks made with lentils, nuts, and seeds are invaluable for PwD. Bengal gram, peanuts, or sesame seeds made into *ladoos* or bars (*chikki*) with jaggery/dates make excellent healthy desserts, especially before, during, and after vigorous play, or at bedtime, to prevent hypoglycemia.

### 4.8. Micronutrients

Children and adolescents with diabetes have the same vitamin and mineral requirements as their peers. Deficiencies impact growth, intelligence, immune function, and wellbeing. The ISPAD 2018 Guidelines emphasize that these needs should be met by consuming a mixed, balanced diet rather than artificial supplements [5]. The Indian Academy of Pediatrics recommends diet diversity with the inclusion of all food groups to reduce micronutrient deficiencies (“hidden hunger”), which are becoming increasingly prevalent in childhood [107,108,109].

Traditional Indian food-processing styles ensured the daily consumption of greens, colored fruits and vegetables, grains, legumes, herbs, and spices [109]. The addition of acidic compounds like gooseberries or tamarind, and cooking in cast iron vessels, enhanced iron absorption. Soaking grains and pulses reduce phosphates and phytates; fermentation improves vitamin B12 content and enhances phytase activity (which reduces anti-nutritional factors such as phytic acid); germination reduces tannins in some legumes; and mild heat treatment (porridges) helps to release bound carotenoids [18,19]. The addition of fat-rich seeds, such as peanuts in *koshimbir* (a type of cucumber salad)*,* improved the absorption of fat-soluble nutrients such as retinol and provitamin-A carotenoids. Adequate calcium, phosphorus, and magnesium intake is ensured by consuming low-fat dairy and fresh, green vegetables. Milk and milk products, finger millet, beans, dark green leafy vegetables, sesame seeds, amaranth seeds, and horse gram are rich sources of calcium. Magnesium is abundant in nuts, seeds, legumes, whole grains, and leafy greens [17]. The bioavailability of zinc, found in meat, seafood, legumes, nuts, seeds, eggs, and whole grains, was increased by heating, soaking, and sprouting. Fresh vegetables and fruits are also sources of antioxidants; orange/ red ones provide carotene. Gooseberry, raw mango, and citrus fruits are rich in vitamin C. Vitamin E is available in sunflower seeds and peanuts [57].

### 4.9. Beverages

Hydration is important for good health and requires extra attention in PwD, particularly during and after exercise, and during periods of poor glycemic control, when polyuria may occur. Current-day commercial sugar-sweetened beverages (SSB), juices (sweetened or unsweetened), and even sugar-free drinks are linked to obesity [110]. High glycemic excursions after consuming SSB are difficult to control and impair glycemic control in diabetes.

The traditional Indian drink of choice was water stored in an earthenware pot. This helped purify and alkalinize the water while cooling it naturally. A wide range of refreshing beverage options were popular, some giving a ‘cooling’ effect in the summer, others were ‘warming’ for the winter. These beverages also provided electrolytes and nutrients.

The impact of these beverages on glycemic control is variable and depends on the composition. Beverages recommended routinely in PwD are low calorie, low carbohydrate fluids, like thin buttermilk, *rasam*, ginger cumin water, unsweetened lemon juice, amongst others [1,2,111]. Some of the common examples are provided in Supplementary Table S4.

### 4.10. Salt

PwD should be as careful about sodium intake as the general population [105]. A range of spices widely used in Indian cooking (cumin, cardamom, lemon, tamarind, *kokum*, vinegar, onion, garlic, and fresh/dry raw mango) commonly replace salt as taste enhancers and therefore reduce the need for salt.

### 4.11. Spices and Other Superfoods

The wide array of spices (*masalas*) used in Indian cooking provides immense flavor and function. Some common Indian spices used in daily cooking are listed in Box 4. Aromatic leaves including curry leaves, mint, and dill, as well as superfoods, such as gooseberry and moringa [112], add flavor and provide health benefits such as antimicrobial, anti-lithogenic, anti-mitogenic, antioxidant, and anti-inflammatory activity [113,114]. Incorporating spices, herbs, and condiments into meals has shown to improve the glucose and lipid profile for diabetes [17].

Box 4 **Common Indian spices used in Indian cooking.**

Turmeric, cumin, black cumin, coriander, saffron, green cardamom, large cardamom, cinnamon, fenugreek seeds, black and white pepper, long pepper, mustard seeds, carom, bay leaves, nutmeg, mace, ginger, cloves, garlic, asafoetida, fennel, dried pomegranate seeds, nigella seeds, dried mango powder, various chillies, star anise

### 4.12. Prebiotics and Probiotics

Prebiotics are non-digestible carbohydrates that gut bacteria feed upon; their importance in maintaining good gut health is now being recognized. As discussed above, traditional Indian diets had several sources of insoluble resistant starches which provide good prebiotic function [115].

Probiotics are live organisms which, when administered in adequate amounts, confer a health benefit to the host including gut functionality, metabolic homeostasis, reducing cholesterol, preventing obesity, amongst others.

Fermented foods are common in the Indian diet and provide abundant probiotics. Fermentation enhances nutrient bioavailability as well as food digestibility, appearance, flavors, and aroma [116,117]. Examples are live-culture yogurt (lactobacillus, acidophilus, thermophiles, and bifidus), buttermilk, fermented vegetables, and sour pickles.

## 5. Other Considerations

### 5.1. Meal Timings

Ancient Indian texts consider food to be a major preventive and therapeutic tool. They prescribe eating proper amounts, at proper timings, sitting on the floor, and eating together as a family to ‘intensify the digestive fire’ [10]. The ISPAD 2018 Guidelines [5] similarly emphasize the importance of meal-time routines with limitations on snacking, to improve dietary quality and optimize glycemic outcomes. Regularity in mealtimes and routines where the child and family sit down and eat together help establish better eating practices and monitoring of food intake. This is associated with better glycemic outcomes.

The *Samhitas* or *Vedas* advise finishing dinner before sunset, avoiding sleeping on a full stomach, along with very early rising (“with or before the sun”), and a short afternoon siesta. This was appropriate for the tropics and was a practical form of intermittent fasting. In communities where schools start early and close by early afternoon, this routine can be emulated by early waking, post-lunch nap, and early night meal, since late dinner or late bedtime have been shown to contribute to obesity [118].

### 5.2. Insulin Treatment Approaches

In children with type 1 diabetes, insulin type and regimens would differ, based on affordability, school timings, and schedule. These may vary from regimens combining once or twice daily intermediate-acting insulin with pre-meal regular insulin before breakfast, lunch, and dinner; to a once-daily long-acting analog with boluses of regular insulin or rapid-acting analog before each meal and large snack; to rapid-acting analog on an insulin pump. The mixed meal in a *thali,* with greens, complex carbohydrates, proteins, and healthy fats, can be adjusted to ensure smooth post-prandial glycemic excursions, which the insulin bolus can handle well. The child using pre-meal regular insulin may experience hypoglycemia after 2–3 h and would benefit from a mid-meal, portioned, moderate carbohydrate snack like fruit or a pulse-based snack (roasted or boiled pulse, pulse pancake etc.). Individuals on rapid-acting insulin could benefit from a mid-meal, portioned, low-carbohydrate snack such as peanuts, nuts and seeds, cottage cheese (stir-fried/grilled/with vegetables), yogurt, buttermilk *raita*, or egg. Many of the Indian snacks and beverages can be prepared in a low-carbohydrate form, which may not need a pre-meal insulin bolus.

### 5.3. Celiac Disease

Celiac disease is more common in people with type 1 diabetes. The diagnosis can be devastating for families, who fear the severe dietary restrictions. The variety of gluten-free options available in traditional Indian food ease the problem. These include rice, millets, maize, pulses, buckwheat, amaranth, and flours of raw banana or jackfruit. Vegetables and fruits, nuts and seeds, spices and chutneys, dairy products, eggs, and other non-vegetarian foods, can all be enjoyed in a gluten-free diet [119].

### 5.4. Festive Foods of India

India celebrates multiple festivals that are associated with festive foods—sweet and savory. These were usually season-specific, mostly using fresh ingredients. Many items have a low GI, and include healthy cereal-pulse combinations, nuts and seeds, pseudocereals, vegetables, and fruits. Preparations with ingredients like *neem* had medicinal qualities [120]. However, dishes high in refined flour, sugar, salt, and fat have crept in. Their portion size must be kept small in a healthy diet. One way of doing this was serving these foods in small portions as offerings (*prasad*), along with moderate GI foods such as spiced gram, peanuts, green gram, or other pulses with coconut. Ripe bananas were also often given as *prasad*: the comparatively higher GI was particularly helpful after fasting. Similar principles can be followed during parties and functions, with wise choices, small portions, and covering extra carbohydrates, protein, and fat with adequate extra insulin and extra physical activity [105].

Some examples of festive foods of India are provided in Supplementary Table S5 [1,121].

## 6. Limitations

This is not a systematic review of the literature. An attempt has been made in this narrative review to revisit traditional Indian food wisdom and healthy ancient culinary practices, discussing how the principles might be useful and applicable in children and adolescents living with type 1 or type 2 diabetes globally. Many common use practices do not have peer-reviewed evidence published to specifically support children with type 1 diabetes.

## 7. Conclusions

Traditional Indian food practices evolved over thousands of years and provide a holistic approach. The emphasis was to provide wholesome, balanced, nourishing meals, which were also visually appealing and palatable. Meal combinations were guided by scientifically sound principles, ensuring nutrient density and diet diversity. The traditional Indian *Thali* matches well with the current ISPAD Clinical Practice Guidelines for type 1 diabetes. Various cooking methods and meal combinations enhance the nutrient bioavailability and lower the GI, thus making it beneficial to both the general population and for people with diabetes. Many of these ancient traditions continue or are being revived. It would be helpful to incorporate these food concepts, combinations, and techniques in our daily lives. Diabetes care teams across the world can help people with diabetes, and their families, explore these options to improve glycemic control and quality of life.

**Figure 2:**
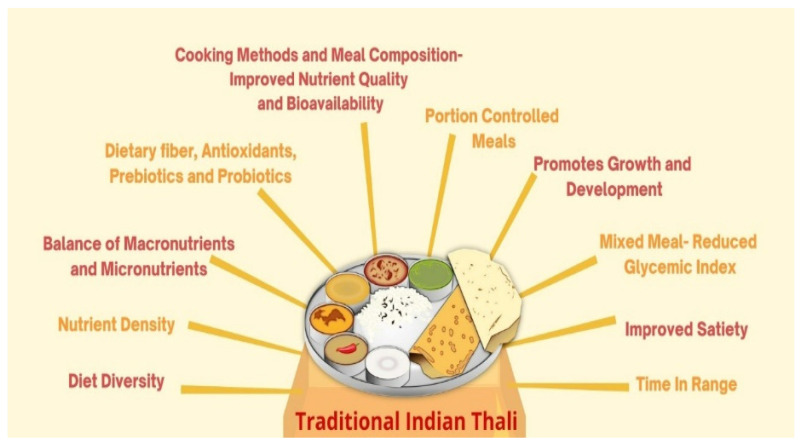
Benefits of the traditional Indian *Thali* in meeting nutritional requirements and achieving glycemic control in people with diabetes (PwD).

Some common, practical, simple suggestions for lowering GI are listed in Box 5. The benefits of the *Thali* is schematically represented in Figure 2.

Box 5 **Traditional ways to lower glycemic index (GI) and improve the nutritive value of meals.**

- Processing grains to the minimum. Using unpolished, coarse, long grain, and aged grains
- Using slow digesting (lente) carbohydrates with higher amylose and soluble fiber content such as pulses and barley
- Using meal combinations to add protein, fiber, and healthy fat to meals
- Using resistant-starch-rich foods and methods which enhance resistant starch content
- Using an acidic medium such as lemon, vinegar, or tamarind
- Using slightly unripe fruits, since the GI increases as fruit ripens

## Figures and Tables

**Table 1:** Methods and techniques used in Indian cooking and its nutritional significance.

Cooking Methods	Nutrition Highlights
Combination of cereal and pulse (*Khichdi/pongal/idli/dosa/dhokla/dal baati/zunka or pitla bhakar/dal dhokli/handvo/thalipeet/puttu kadala/dal paratha*)	Cereals and pulses, when combined in the ratio of 3:1, complement one another, providing complete protein with all essential amino acids [15,16] Decrease GI of meals [17]
Fermentation (*idli/dosa/dhokla/enduri pitha/curd/selroti/ambeli/khaman/sez/adai/vada*) *	This produces beneficial enzymes which aid in digestion.The microorganisms produce vitamin B12 and β-galactosidase: the enhanced probiotic activity helps maintain gut health and improves immunity. Fermentation of pulses reduces anti-nutritional factors such as phytic acid, thereby increasing nutrient availability. Propensity to flatulence is decreased.Overnight fermentation of *idli* and *dosa* batter increases the vitamins B and C content. Fermentation of milk into yogurt converts lactose into lactic acid: beneficial for those with lactose intolerance [18,19].
Cooking and cooling starchy foods	Retrogradation (re-crystallization of starch polymer chains, which occurs after the gelatinization) increases the resistant starch type 3 (RS 3) content, reducing the GI. RS3 is formed when starchy foods are cooked, cooled and stored for several hours. Cooked and cooled potatoes, rice and legumes have been shown to contain significant amounts of RS3 [20,21,22,23]
Soaking (pulses and cereals)	Soaking before cooking reduces cooking time, improves digestibility, and reduces anti-nutritional factors like phytates, tannins and enzyme inhibitors [15,24,25].
Steaming (vegetables)	One of the best cooking methods for preserving nutrients, including water-soluble vitamins that are sensitive to heat and water, like Vitamin C and B complex, and phytochemicals [24,25,26]
Sand Roasting (Popped, puffed, and flaked rice and maize; roasted cereals and millets; roasted legumes such as groundnut, chickpea, pea, cowpea)	It is the simplest, most inexpensive method of dry heat application. High-temperature short-time treatment in sand results in higher puffing, crispiness, volume, improved color, aroma, flavor, and texture while enhancing shelf life. The sand roasting process enhances carbohydrate and protein digestibility, β-glucan extractability, levels of prebiotic dietary fiber, minerals, and antioxidants; and reduces the inherent anti-nutrient levels in cereals and legumes. The destruction of seed microflora enhances the shelf life and consumer acceptance. These roasted rice, wheat, maize, millets and legume preparations are healthy alternatives to current-day unhealthy snacks [27]
Soaking and fermenting cooked rice overnight (*pakhala, panta bhaat*)	This process increases vitamin B6 and B12 content. This process also increases beneficial bacteria, which helps in digestion, improves gut health and boosts immunity [28].
Pickling (vegetables, fruits, fish, chicken, meat)	Pickling, one of the oldest methods of preservation, imparts unique and desirable changes in flavor, texture and color. It also increases the probiotic potential [29].
Sprouting (green gram, chickpeas, Bengal gram, other legumes)	Sprouting increases the content of vitamin C and some B-group vitamins. This also reduces phytic acid and enhances the absorption of zinc. This also enriches vitamin A [25].
Cooking in earthen pots	Traditionally, Indian households cooked in earthen and iron pots. Benefits include the ability of earthen vessels to absorb moisture (due to their porous nature) and let heat circulate evenly and slowly through the food being cooked, making it aromatic and retaining nutrition. Clay being alkaline balances the pH and neutralizes acidity. It adds many important nutrients like calcium, magnesium, iron and phosphorus. Cooking in a clay pot needs much less oil [28,30]. (Clay pots available today are often glazed with substances containing lead, mercury and others which are hazardous for health. Unglazed, pure earthen pots are used for cooking, after soaking in water for a few hours)
Cooking in iron pots	It is a useful method of iron fortification and prevents iron-deficiency anemia [30,31]

References [15,16,18,19,20,21,22,23,24,25,26,27,28,29,30,31]. * The local names of the individual food items which are prepared by the cooking methods are italicized here. Details of many of these preparations are available in the Supplementary Materials.

## Supplementary Materials

The following are available online at https://www.mdpi.com/article/10.3390/nu13124427/s1, Table S1: Common Indian preparations with grains that increase nutritive value and lower glycemic index (GI), Table S2: Some common Indian preparations with pulses with high nutritive value and low glycemic index (GI), Table S3: Some common Indian recipes with cereal pulse combinations, Table S4: Some examples of commonly consumed beverages of India, Table S5: Some examples of festive foods of India.
