# Supplementary material for: ‘Old Is Gold’: How Traditional Indian Dietary Practices Can Support Pediatric Diabetes Management
Source: Nutrients. 2021 Dec 10;13(12):4427. doi: 10.3390/nu13124427 (PMC8707693; doi:10.3390/nu13124427)
Supplement: Supplementary file 1 [file nutrients-13-04427-s001.zip › nutrients-1454265-supplementary.pdf]

Supplementary Table S1: Common Indian preparations with grains that increase nutritive value and lower glycemic index (GI) [1].

Recipe	Method of preparation
Curd Rice	Curd (yogurt) added to cooked rice; tempered with peanuts, curry leaves, mustard seeds and asoetifida in a little oil
Lemon Rice	Lemon juice squeezed onto cooked rice; tempered with peanuts, other nuts, curry leaves, turmeric, mustard seeds and asafoetida in a little oil
Tamarind Rice	Tamarind pulp added to cooked rice; tempered with peanuts, other nuts, pomegranate, curry leaves, turmeric, mustard seeds and asafoetida in a little oil
Tomato Rice	Rice cooked with tomatoes, onions, garlic, curry leaves, and asafoetida
Masale Bhaat/ Podnichi bhaat	Leftover rice cooked with vegetables; tempered with peanuts, curry leaves, turmeric, mustard seeds and asafoetida in a little oil
Vegetable Pulao	Rice cooked with vegetables and spices
Coconut Rice	Grated coconut and roasted nuts added to tempered cooked rice
Pakhala/ pazhaya saadam/ paniwala chawal/ panta bhaat	Cooked rice soaked in plenty of water and fermented overnight; spiced with curd/lemon, salt, mint leaves, crushed ginger; consumed along with the superfluent water
Biryani	Rice slow cooked with vegetables/ meat/ egg/ fish/ cottage cheese, and fragrantly spiced
Poha/ Chuda Upma	Soaked rice flakes cooked with vegetables, sprouts, peanuts, turmeric, and onions; with fresh lemon juice squeezed on top
Dadpe Pohe	Raw poha or flattened rice mixed with chopped onions, tomatoes, grated carrots; moistened with juices from the vegetables; then tempered with mustard seeds, asafoetida, peanuts and curry leaves in a little oil
Chuda Dahi	Soaked rice flakes mixed with curd, cottage cheese, grated coconut, jaggery
Rice *Seviyan*	Rice vermicelli cooked with vegetables, peanuts; tempered with mustard seeds, and curry leaves in a little oil.
Bhel/ Jhalmuri	Puffed rice with peanuts, onion, tomatoes, chillies, mashed potato, roasted split bengal gram, coriander leaves
Rice Puttu	Rice flour steamed with grated fresh coconut
Appam	Pancakes with rice and coconut batter
Poha chivda	Roasted rice flakes tempered with mustard, curry leaves and peanuts
Panki	Batter of rice flour, curd and spices spread between banana leaves and steamed
Phodnichi poli/ Roti churma/ Seyal mani	Leftover *chapatis* crushed and tempered with spices, vegetables and peanuts

Semolina *upma*	Salted thick porridge of wheat semolina with vegetables, split dals and nuts
Broken wheat *upma*	Salted thick porridge of broken wheat with vegetables, split dals and nuts
Roti nu shak	Leftover chapatis cooked with garlic and buttermilk
Stuffed *parathas*	Vegetables, cottage cheese or minced meats stuffed in whole wheat flour dough, rolled into *chapati* like flatbreads, then cooked with oil on a griddle
Thepla	Thin flatbread made from fresh fenugreek leaves and whole-grain flours (mixture of whole wheat and gram flour)
Kathi rolls	Skewered meats, or cottage cheese, or vegetable-sprouts mixture, wrapped in a *paratha*
Egg roll	Egg wraps (wrap made of whole wheat flour)
Muthiya	Steamed dumplings made using whole wheat flour, chickpea flour, fenugreek leaves, grated bottle gourd, salt, turmeric, chili powder; tempered with seasoning
Millet *idli*	Steamed millet cakes made with fermented batter of rice and whole pulses in the ratio 3:1; eaten with pulse & vegetable curry *(sambhar)* and coconut/ roasted dal chutney with spices and ghee
Millet *upma*	Salted thick porridge of millet with vegetables, split dals and nuts
Millet *chapati*	Flatbread made from millets
Bajra millet koozh	Pearl millet flour cooked with chopped shallots and buttermilk
Bajre ki khatte raabdi	Pearl millet flour mixed with curd; cooked with spices
Gujarati Methi Na Dhebra	Multigrain millet *paratha* made from sorghum and pearl millet; flavoured with fenugreek leaves and spices.

Supplementary Table S2: Some common Indian preparations with pulses with high nutritive value and low glycemic index (GI)

**Recipe**	**Method of preparation**
Dal	Pulse curry tempered with spices
Sambhar	Pulse-based vegetable curry cooked with pigeon pea and tamarind
Dahi Kadhi	Savoury vegetable curry made with gram flour and curd gravy
Sindhi Kadhi	Gravy made from chickpea flour, vegetables and tamarind pulp
Pakoras	Deep fried sliced vegetables/ nuts/ *paneer*/ meat/ fish after coating in a thick spiced up gram flour batter
Khaman dhokla	Steamed savoury cakes made from fermented gram flour and curd batter
Khandvi	Steamed savoury rolls made from fermented gram flour and curd batter
Gatte ki sabji	Gram flour roundels added to a curd-based gravy
Papad	Green gram and black gram flour rolled into thin spiced and seasoned discs, dried and stored. Freshly roasted or fried before consuming as an accompaniment with food
Chana jor, moong jor	Spiced, flattened and roasted black gram, chickpea or green gram
Chana chaat, ghoogni and ragda	Boiled dried white peas or gram seasoned with spices; and added onion, tomatoes and raw mango
Dhansak/ dal ghosht	Pulses cooked with vegetables and/ or sometimes meat
Dalma	Pulse cooked with vegetables, raw papaya and spices
Kootu	Pulse and vegetable (bottle gourd, pumpkin, carrot, cabbage) curry
Saag dal	Pulse and green leafy vegetable

Supplementary Table S3: Some common Indian recipes with cereal pulse combinations

**Recipe**	**Method of preparation**
Vegetable khichdi	Rice/ millets and pulses cooked together with vegetables
Pongal	Rice/ millets and pulses cooked together with vegetables
Idli	Steamed rice/ millet cakes made with fermented batter of rice and whole pulses in the ratio 3:1; eaten with pulse vegetable curry (*sambhar*) and coconut/ dal *chutney* with spices and ghee
Dosa	Crepes made with fermented batter of rice/ millets and whole pulses in the ratio 3:1; eaten with *sambhar* and coconut/ dal *chutney* with spices and ghee
Paniyaaram	Ball shaped dumplings made by steaming fermented batter of cereal/ millet flour and ground pulses using a mould
Dhokla	Steamed cakes made with fermented batter of rice/ millets, pulse flour and sour curd. The cakes are tempered with curry leaves and mustard seeds
Dal baati	Pulse curry consumed with roasted whole wheat flour balls
Zunka or pitla bhakar	Chickpea flour or horse gram flour gravy consumed with rice/ millet flour *rotis*
Handvo	Steamed savoury cake made using rice, pulses and vegetables.
Dal dhokli	Pulse curry cooked with raw whole wheat flour *chapati*, peanuts and vegetables
Thalipeet	Flatbread made from whole-grain flours (rice, millets) and pulses with onions
Puttu kadala	Steamed rice/ millet flour and coconut log consumed with *kadala kari* (black chickpea curry)
Dal or Sattu paratha	Wheat flour dough is stuffed with sattu/ roasted chickpea flour, onion, grated ginger and green chillies
Dal Rice/ Sambhar rice/ Pulse curry rice (Rajma rice or chole rice)	Cooked pulse curry consumed with steamed rice
Bisi bele bhaath	Rice and pulses cooked with vegetables, mainly carrot, beans and various spices
Dhirde	Pancake made from cereals/ millets and pulses or one grain/legume, onion, tomatoes, turmeric powder, cumin seeds, green chilies, salt, coriander leaves

Supplementary Table S4: Some examples of commonly consumed beverages of India [1-3]

**Beverage and preparation method**	**Nutritional highlights**	**Special mention**
Jal jeera: Roasted cumin powder, ginger, black pepper powder, salt and mint, in water	Stimulates appetite<br>Provides cooling effect<br>Is a good digestive	
Aam Pora: Roasted raw mango pulp, roasted cumin powder, sugar, mint leaves, salt, in water	Good appetizer.<br>Glucose lowering properties	Added sugar must be minimized
Sattu drink: *Sattu* (roasted chickpea/ barley flour), roasted cumin powder, black salt, lemon in water.	Low GI<br>Rich in protein, fiber, minerals	
Salted *Lassi*: Curd, roasted cumin powder, mint and coriander leaves, salt, blended in water.	Good probiotic<br>Aids digestion<br>Good thirst quencher	Avoid sweet *lassi*
Kanji: Black carrots, spiced with ground mustard seeds, red chilli powder, black salt. All ingredients mixed with boiled and cooled water, kept in the sunshine in a glass jar for 2-4 days for fermentation.	Low GI<br>Natural probiotic<br>Rich in antioxidants.<br>Helps correct hyponatremia in the summer	
Nannari: Root extracts of *Decalepis hamiltonii* blended with lime, in water	Aid in metabolism<br>Rich in antioxidants	
Tender coconut water	Natural electrolyte source<br>Rich in potassium<br>Helps maintain hydration	Consume when BG well controlled
Kashmiri *Kahwa*: Green tea, cardamom, cinnamon, saffron, almond slivers boiled in water.	Provides warmth in winter.<br>Excellent beverage to soothe the nerves<br>Aids digestive health	Avoid/ minimize added sugar
Torani: Superfluent water from overnight soaked cooked rice *(pakhala)*	Natural probiotic drink<br>Ensures hydration<br>Provides essential minerals and nutrients	
Rasam: Soup made with dal water, tomato and spices	Good remedy for common cold and viral infections<br>Low GI, low carbohydrates, low calories.	

Supplementary Table S5: Some examples of festive foods of India [1,4,5]

Food Items	Nutritional Highlights	Proposed Modification
Mahaprasad: Green gram, black chickpeas & unpolished rice soaked overnight; mixed with fresh coconut, salt, sugar & fresh seasonal fruits	Good source of resistant starch & medium chain fatty acids	Carbohydrate content and GI can be lowered by exclusion of sugar
Bevubella or *Ugadi Pachadi*: Mixture of neem flowers, grated raw mango, tamarind, pepper powder, jaggery, salt	Neem contains several chemical compounds like epoxyazadiradine (lowers BG), flavonoids, tannins, galic acid etc. which provide antioxidant effects	Amount of jaggery can be reduced to lower GI and carb content.
Khichuri and *Labda*: Porridges made with unpolished rice, yellow lentils, cumin, salt, and different types of vegetables.	Complete, wholesome, nutritious meal<br>Rich in fiber, good quality protein, micronutrients, polyphenolic compounds, and antioxidants.<br>GI kept low by added lentils and vegetables	
Jil Pitha: Chicken curry mixed with rice flour into a paste, spread uniformly on sal leaves, carefully sealed with neem twigs, and steamed in a mud vessel. The leaves are removed before serving.	Protein rich<br>Nutrients preserved by steam cooking.	
Shirni: Milk, sugar, whole wheat flour, banana blended well; served with added nuts.	Quickly, easily made.<br>Added nuts provide healthy fats.	Sugar can be excluded;<br>Low fat milk helps reduce calorie count
Dodhikorma: Popped rice (*khoi*), flattened rice (*poha*), sweetened curd (*mishti doi*), curd, sugar drops (*batasha*), sweets (*sandesh/ rasogolla*), sugar, ripe banana, shredded coconut, cashew nuts, raisins, dates, seasonal fruit mixed together, made into big *ladoos*	Complete, wholesome, nutritious recipe<br>Rich in good quality protein, carbohydrates and fats.	Sugar can be excluded<br>Proportion of sweets, raisins, sweet curd, and ripe banana can be reduced to lower carb content and GI
Nombu Kanji: Porridge made from broken rice/rice grits, yellow lentil, vegetables, mutton/ chicken (optional), fenugreek seeds, grated coconut/ coconut milk, onion, tomato, spices and green chillies	Complete, wholesome meal<br>Packed with macro- and micronutrients<br>Low GI<br>Improve gut health	Can be modified by adding seasonal vegetables, and drumstick leaves which enhance nutritional value

Gustaba: Minced mutton mixed with aniseed & ginger powder, salt, rolled into balls and steamed. The steamed balls are cooked in a gravy of curd, garlic, cinnamon, cardamom, onion and *ghee*	Good source of protein & minerals.<br>In cold climate, helps keep the body warm	Nutritional value can be enhanced with addition of vegetables.

1. Nambiar, V. *Indian Food Anthropology and The Eat Right Movement.* , 1st ed.; Selective & Scientific Books: 2021.
2. Evans, S.F.; Meister, M.; Mahmood, M.; Eldoumi, H.; Peterson, S.; Perkins-Veazie, P.; Clarke, S.L.; Payton, M.; Smith, B.J.; Lucas, E.A. Mango supplementation improves blood glucose in obese individuals. *Nutr Metab Insights* **2014**, *7*, NMI. S17028.
3. Sarkar, P.; DH, L.K.; Dhumal, C.; Panigrahi, S.S.; Choudhary, R. Traditional and ayurvedic foods of Indian origin. *Journal of Ethnic Foods* **2015**, *2*, 97-109.
4. Islas, J.F.; Acosta, E.; G-Buentello, Z.; Delgado-Gallegos, J.L.; Moreno-Treviño, M.G.; Escalante, B.; Moreno-Cuevas, J.E. An overview of Neem (Azadirachta indica) and its potential impact on health. *Journal of Functional Foods* **2020**, *74*, 104171, doi:https://doi.org/10.1016/j.jff.2020.104171.
5. Ganesan, K.; Xu, B. Polyphenol-Rich Lentils and Their Health Promoting Effects. *International journal of molecular sciences* **2017**, *18*, doi:10.3390/ijms18112390.
